# An equation to estimate the difference between theoretically predicted and SDS PAGE-displayed molecular weights for an acidic peptide

**DOI:** 10.1038/srep13370

**Published:** 2015-08-27

**Authors:** Yihong Guan, Qinfang Zhu, Delai Huang, Shuyi Zhao, Li Jan Lo, Jinrong Peng

**Affiliations:** 1 MOE Key Laboratory for Molecular Animal Nutrition, College of Animal Sciences, Zhejiang University, 866 Yu Hang Tang Road, Hangzhou, 310058 China

## Abstract

The molecular weight (MW) of a protein can be predicted based on its amino acids (AA) composition. However, in many cases a non-chemically modified protein shows an SDS PAGE-displayed MW larger than its predicted size. Some reports linked this fact to high content of acidic AA in the protein. However, the exact relationship between the acidic AA composition and the SDS PAGE-displayed MW is not established. Zebrafish nucleolar protein Def is composed of 753 AA and shows an SDS PAGE-displayed MW approximately 13 kDa larger than its predicted MW. The first 188 AA in Def is defined by a glutamate-rich region containing ~35.6% of acidic AA. In this report, we analyzed the relationship between the SDS PAGE-displayed MW of thirteen peptides derived from Def and the AA composition in each peptide. We found that the difference between the predicted and SDS PAGE-displayed MW showed a linear correlation with the percentage of acidic AA that fits the equation *y* = *276.5x* *−* *31.33* (*x* represents the percentage of acidic AA, 11.4% ≤ *x* ≤ 51.1%; *y* represents the average ΔMW per AA). We demonstrated that this equation could be applied to predict the SDS PAGE-displayed MW for thirteen different natural acidic proteins.

Digestive organ expansion factor (Def) is a nucleolar protein that is conserved across the kingdoms including yeast, *Drosophila, Arabidopsis*, zebrafish, mouse and human^1^,^2^,^3^. In zebrafish, loss-of-function of Def (*def*^*−/−*^) caused hypoplastic digestive organs due to cell cycle arrest but not to apoptosis^1^. This outcome is attributed to the stabilization of the p53 protein in *def*^*−/−*^ that in turn directly transactivates the expression of p53 isoform Δ113p53 whose function is to antagonize p53 apoptotic activity^2^,^4^,^5^. Strikingly, the stabilized p53 in *def*^*−/−*^ is accumulated in the nucleolus^2^. Recently, it was found that Def complexes with a cysteine protease Calpain 3 to degrade p53 in the nucleolus^2^. Further investigation showed that Def haploinsufficiency in *def*^+/−^ heterozygous fish activates p53-dependent TGFβ signaling pathway and leads to scar formation after partial hepatectomy^6^. In addition to its role in regulating p53 function, Def is also involved in pre-rRNA processing in different organisms^3^,^7^,^8^,^9^.

However, during our previous work we were puzzled by one observation that Def run a band of MW ~100 kDa on an SDS PAGE gel, that is approximately 13 kDa larger than its theoretically predicted MW (86.8 kDa). To find out the cause for this size discrepancy, we carried detailed characterization of the Def protein by various means. We first ruled out the possible contribution of glycosylation or ubiquitination/sumoylation modification to this observed MW difference by demonstrating that Def lacks such chemical modifications. Def is an acidic protein with a predicted isoelectric point of 5.2. In some reports it was discussed that high content of acidic AA might affect the gel mobility shift of a protein^10^,^11^,^12^,^13^,^14^,^15^. Through analyzing thirteen different peptides derived from Def we finally established that the difference between the predicted and SDS PAGE-displayed MW showed a linear correlation with the contents of acidic AA, i.e glutamate (E) and aspartate (D), that fits the equation *y* = *276.5x* − *31.33* (where *x* represents the percentage of E and D, 11.4% ≤ *x* ≤ 51.1%, and *y* represents the average ΔMW per AA residue).

## Results

### The N-terminus of Def causes the difference between the predicted and SDS PAGE-displayed MW

**Figure 1:**
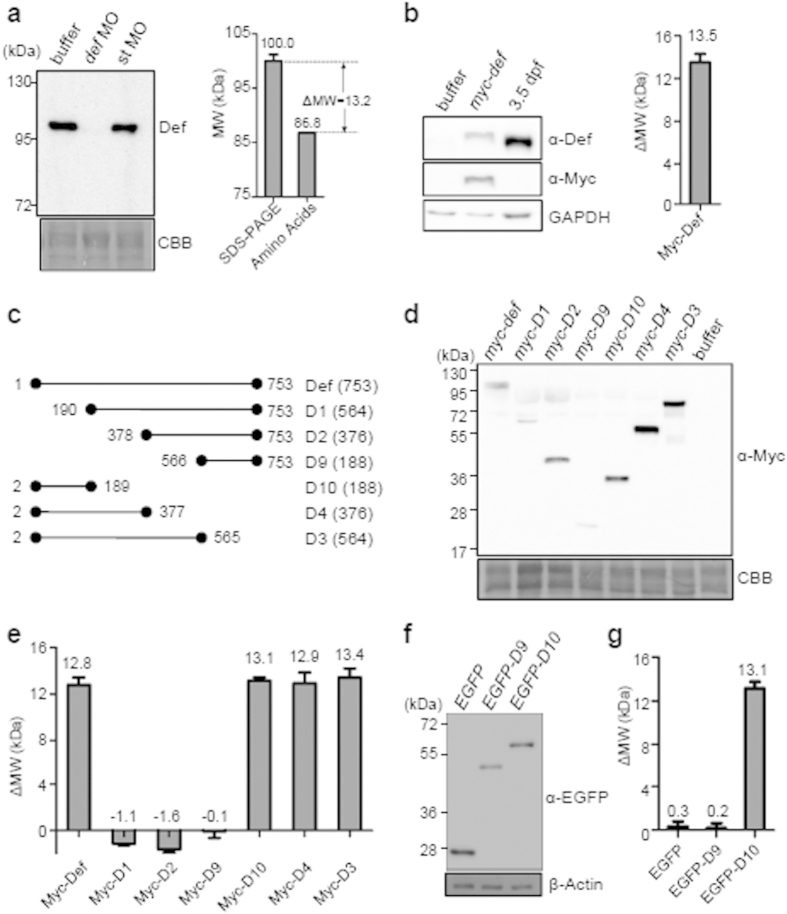
Def N-terminus causes the difference between the predicted MW and the SDS PAGE-displayed MW. (**a**) Western blot of endogenous Def in 3.5 dpf zebrafish embryos (left panel). One-cell stage embryos were injected with buffer, *def*-MO (*def* morpholino) or st-MO (standard control morpholino). Comparison of the SDS PAGE-displayed and the predicted Def MW (right panel). ΔMW = MW^SDS−PAGE^ − MW^predicted^. (**b**) Western blot of Myc-tagged Def in the embryos injected with mRNA at 8 hpf and of endogenous Def at 3.5 dpf (left panel). Right panel: ΔMW = MW^SDS−PAGE^ − MW^predicted^. (**c**) Diagram showing different Def deletions. Numeration denotes the position of amino acid residue. Number of amino acid residues in each peptide is shown in brackets. (**d**) Western blot using the anti-Myc antibody to detect Myc-tagged Def and its derivatives in embryos eight hours after injection with their respective mRNA. (**e**) ΔMW (MW^SDS−PAGE^ − MW^predicted^) for Myc-Def and each of the Myc-tagged Def derivatives calculated based on **d**. (**f**) Western blot using the anti-EGFP antibody to detect EGFP, EGFP-D9 and EGFP-D10 in embryos eight hours after injection with their respective mRNA. (**g**) ΔMW for EGFP, EGFP-D9 and EGFP-D10 based on **f**. Loading control: CBB (coomassie brilliant blue) staining or western blot of GAPDH or β-Actin. In **a** (n = 3), **b** (n = 3), **e** (n = 3) and **g** (n = 4), value above indicates the ΔMW mean and error bar stands for SEM. The gel picture (for CBB staining) and western blot images were cropped with a grey cropping line. All gels for western blot analysis were run under the same experimental conditions.

The nucleolar protein Def in zebrafish is composed of 753 AA with a predicted MW of approximately 86.8 kDa. However, we noticed that, in western blot, endogenous Def protein migrated as a protein of approximately 100.0 kDa in an SDS-PAGE gel (Fig. 1a), approximately 13 kDa larger than the predicted MW (Fig. 1a). We fused a Myc-tag in-frame to the 5′-end of *def* full length cDNA and injected the *myc-def* mRNA into one-cell stage wild type embryos. Western blot result showed that the expressed Myc-Def was also approximately 13 kDa larger than the MW predicted for Myc-Def (Fig. 1b). To determine whether there is a specific region/domain in Def that caused this MW difference (ΔMW) we generated six different Def deletion fragments and fused them with a Myc-tag in the expression vector pCS2^+^, respectively (Fig. 1c). We then injected the mRNA corresponding to each of these Def deletion constructs to one-cell stage embryos and analyzed the sizes of their protein products. The result showed that the three N-terminal deleted Def protein products (D1, D2 and D9) did not exhibit drastic variations from their predicted MW (Fig. 1c–e). In contrast, the C-terminal deleted Def products (D3, D4 and D10) all exhibited a MW size that is approximately 13 kDa larger than their predicted MW (Fig. 1c–e). Since D10 is the shortest among D3, D4 and D10 it is logical to make the assumption that the N-terminal 188 (2-189) amino acids is responsible for the observed MW difference. To further confirm this assumption we replaced the Myc-tag in D9 and D10 with EGFP, respectively, and found that the EGFP-D10 fusion protein, but not the EGFP-D9, displayed a size that was approximately 13 kDa larger than its predicted MW (Fig. 1f,g). Therefore, we conclude that the N-terminus of Def houses certain feature that causes the difference between the theoretically predicted and SDS PAGE-displayed MW.

### The N-terminus of Def is not modified by glycosylation

**Figure 2:**
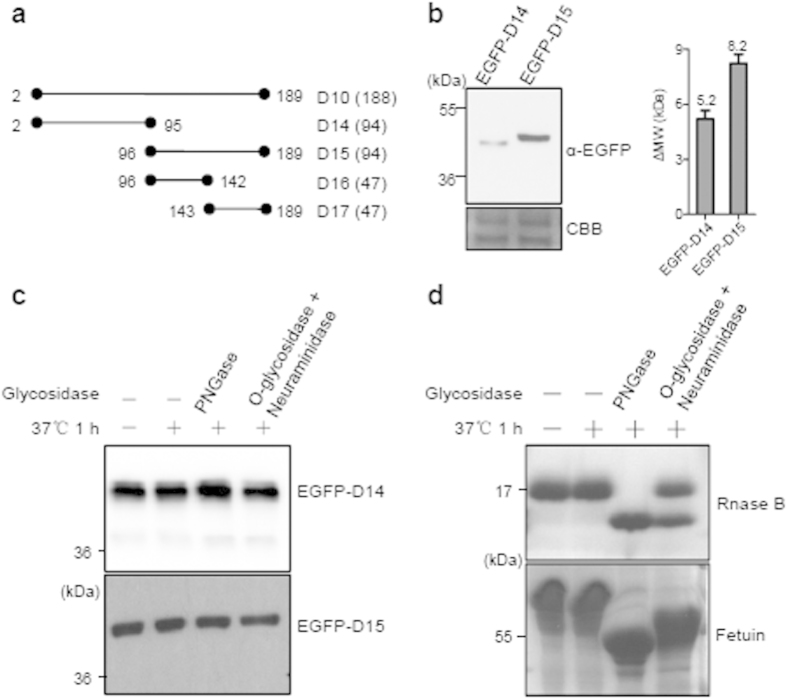
Def N-terminus is not modified by glycosylation. (**a**) Diagram showing different Def derivatives. Numeration denotes the position of amino acid residue. Number of amino acid residues in each peptide is shown in brackets. (**b**) Western blot using anti-EGFP antibody to detect EGFP-D14 and EGFP-D15 (left panel). Right panel shows the ΔMW (MW^SDS−PAGE^ − MW^predicted^) for EGFP-D14 and EGFP-D15. (**c**) Western blot using an EGFP antibody to detect EGFP-D14 and EGFP-D15 treated with or without PNGase or O-glycosidase plus Neuraminidase. Proteins samples were extracted from embryos at 8 hpf after *EGFP-D14* or *EGFP-D15* mRNA injection into one-cell stage embryos. (**d**) Rnase B and Fetuin were used as the positive controls in glycosidase treatment as indicated and were stained with CBB (coomassie brilliant blue). The gel picture (for CBB staining) and western blot images were cropped with a grey cropping line. All gels for western blot analysis were run under the same experimental conditions.

We previously showed that the N-terminus (1-377 AA) of Def is essential for the Def-Capn3 pathway in mediating p53 degradation in the nucleolus^2^. Considering the fact that the N-terminus of Def is also responsible for the MW difference, we were intrigued to study the characteristics of the N-terminus of Def. Considering the ΔMW is ~13 kDa, we first went about finding out whether the discrepancy between the predicted Def MW and its SDS PAGE-displayed MW was attributed to post-translational modification(s), such as glycosylation or ubiquitination, which often causes drastic gel mobility shift^16^,^17^. To facilitate our study, we divided D10 (encoding 188 amino acid residues) by halving it into two parts, namely D14 and D15 (Fig. 2a) and fused them to the EGFP tag. Western blot analysis revealed that the SDS PAGE-displayed MWs for EGFP-D14 and EGFP-D15 were approximately 5.3 kDa and 7.9 kDa larger than their predicted MW, respectively (Fig. 2b). Since N- or O-glycosylation often drastically increases the MW of a protein we treated EGFP-D14 and EGFP-D15 with PNGase (an N-glycosidase, for N-deglycosylation) and O-glycosidase plus Neuraminidase (for O-deglycosylation), respectively. The gel mobility of EGFP-D14 and EGFP-D15 were not affected by treatment with any of these glycosidase (Fig. 2c) whilst, as expected, RNase B and Fetuin, two positive controls, migrated faster after glycosidase treatment (Fig. 2d). Therefore, the N-terminus of Def is not glycosylated and this rules out the possible contribution of glycosylation to the observed MW difference.

### The N-terminus of Def is not modified by ubiquitination/sumoylation

**Figure 3:**
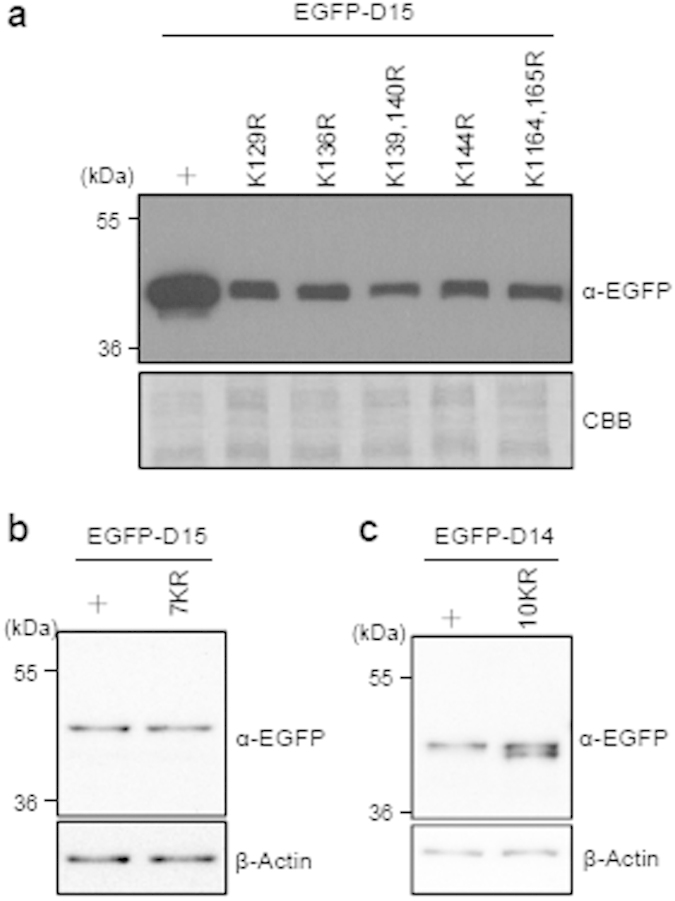
The MW difference is not caused by K modification in D14 and D15. (**a–c**) Western blot using EGFP antibody to detect EGFP-D15 and single or double K to R mutants of EGFP-D15 (**a**) or EGFP-D15_7KR (all seven K in D15 were mutated to R) (**b**) or EGFP-D14_10KR (all 10 K in D14 were mutated to R) (**c**). Protein samples were extracted from embryos at 8 hpf after corresponding mRNA injection into one-cell stage embryos. CBB staining: loading control. The gel picture (for CBB staining) and western blot images were cropped with a grey cropping line. All gels for western blot analysis were run under the same experimental conditions.

Lysine (Lys or K) is an important amino acid for post-translational modifications including methylation, acetylation, sumoylation and ubiquitination^18^. Since D15 exhibited a MW that is ~8 kDa larger than its predicted MW (Fig. 2b) we wondered whether there is(are) any modification(s) on the K residues. In total there are seven K residues in D15. We substituted these K with R singly (K129R; K136R; K144R) or in combination (K139, 140R; K164, 165R) or in summation (all seven K with R, 7KR) in the EGFP-D15 plasmid by site-directed mutagenesis and found that none of the mutant proteins exhibited an obvious gel mobility shift (Fig. 3a,b), thus excluding the possible contribution of lysine modification to the observed MW difference of D15. We also mutated all ten K in D14 and found that the EGFP-D14_10KR protein displayed two bands, with one showing identical MW to the wild type D14 peptide and the other ~1 kDa smaller than the wild type D14 peptide (Fig. 3c). Although currently we cannot explain this observation, considering the fact that this lower band is still about 4 kDa larger than the predicted D14 MW we conclude that the difference between the predicted MW and the SDS PAGE-displayed MW is not caused by K modification in D14.

### High percentage of acidic AA in the N-terminus of Def is the key determinant of the observed MW discrepancy

**Figure 4:**
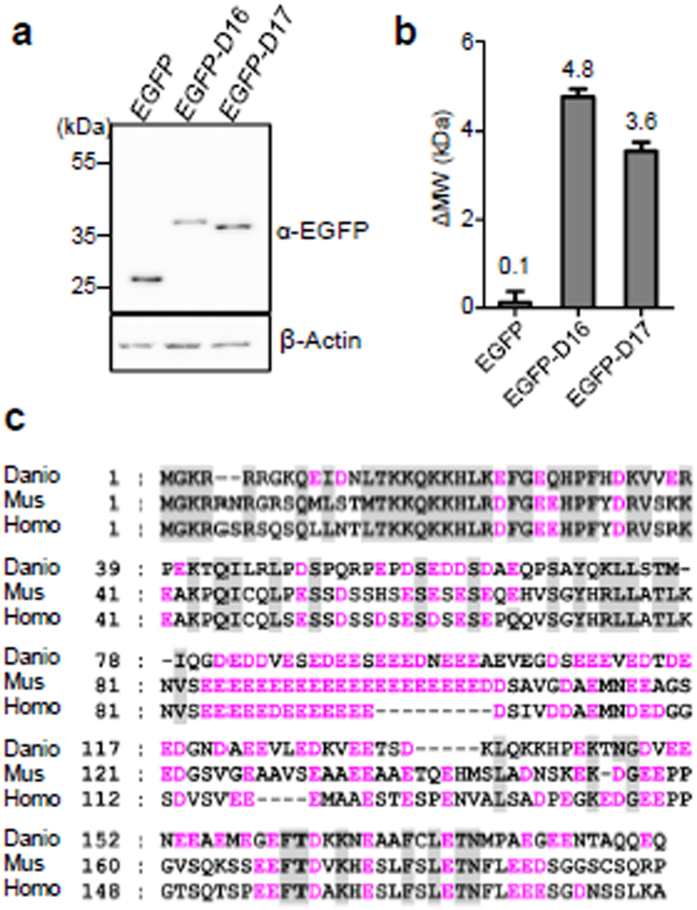
The N-terminus of Def encompasses a high content of E and D. (**a**) Western blot using anti-EGFP antibody to detect EGFP, EGFP-D16 and EGFP-D17. (**b**) Showing the ΔMW (MW^SDS−PAGE^ − MW^predicted^) for EGFP-D16 and EGFP-D17 (n = 4). Value above represents the mean ΔMW and error bar stands for SEM. (**c**) Amino acid sequence alignment of D10 fragment of *Danio rerio* Def (NP_775380.1) and its homologous region in *Mus musculus* (NP_663390.2) and *Homo sapiens* (NP_055203.4). E and D are in pink. Western blot images were cropped with a grey cropping line. All gels for western blot analysis were run under the same experimental conditions.

**Figure 5:**
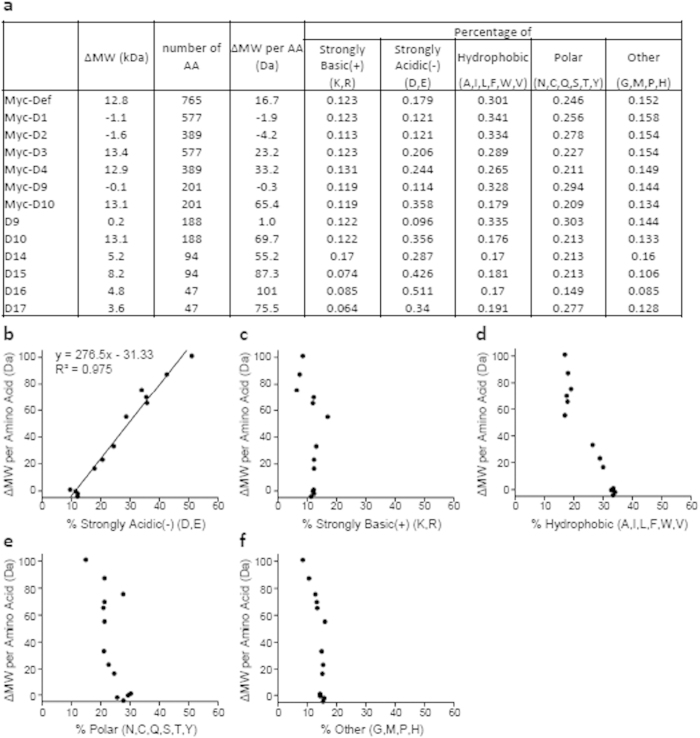
ΔMW shows a linear correlation with the content of acidic AA in a peptide. (**a**) Showing the ΔMW, the number of AA residues and the average ΔMW per amino acid residue in each peptide. The 20 AA were divided into five groups including hydrophobic group (A, I, L, F, W, V), polar group (N, C, Q, S, T, Y), strongly basic group (K, R), strongly acidic group (E, D) and the group of the rest AA (G, M, P, H). The percentages of each of this group in peptides were calculated. (**b–f**) Average ΔMW per amino acid residue in a peptide in an SDS-PAGE gel was plotted against the percentage of strongly acidic AA (E and D) (**b**), strongly basic AA (**c**), hydrophobic AA (**d**), polar AA (**e**) and other AA (**f**). Average ΔMW per amino acid residue on an SDS-PAGE gel is only linearly correlated with the percentage of strongly acidic AA (**b**).

Size analysis showed that both D14 and D15 fragments (Fig. 2b) contributed to the SDS PAGE-displayed MW of D10 (Fig. 1e). We further divided D15 into D16 and D17 and fused them to EGFP, respectively, and found that both EGFP-D16 and EGFP-D17 showed obvious difference between the predicted and SDS PAGE-displayed MW (Fig. 4a,b). Previous reports have implicated that high percentage of acidic amino acid residues might result in retardation of protein mobility^10^,^11^,^12^,^13^,^14^,^15^. Domain analysis showed that the N-terminus of Def contains a glutamate-rich region (amino acid residues 82-206) (Fig. 4c)^2^. We thus went to determine the relationship between the SDS PAGE-displayed MW and amino acid composition of Def. We divided the 20 AA into five groups including hydrophobic (A, I, L, F, W, V), polar (N, C, Q, S, T, Y), strongly basic (K, R), strongly acidic (E, D) groups and a group of the remaining amino acids (G, M, P, H). We then calculated the percentage of each of this group AA in each peptide including Myc-Def, Myc-D1, Myc-D2, Myc-D3, Myc-D4, Myc-D9, Myc-D10, D9, D10, D14, D15, D16 and D17. We also calculated ΔMW for each of the aforementioned peptide and used this ΔMW to divide the number of amino acids to get the average ΔMW per amino acid residue in each peptide (Fig. 5a). We then plotted the percentage of each of the five groups against the ΔMW per amino acid residue in each peptide (Fig. 5b–f). We found that only the percentage of the group of strongly acidic AA showed a linear correlation with the average ΔMW per amino acid residue (Fig. 5b) while none of the other four groups showed such correlation (Fig. 5c–f). A mathematic calculation allowed us to get the linear equation as *y* = *276.5x* − *31.33*, where *x* stands for the percentage of strongly acidic amino acids (11.4% ≤ *x* ≤ 51.1%) and *y* for the average ΔMW per amino acid residue (Fig. 5b). Considering the possible effect of positively charged amino acids (K and R) on the equation, we noticed that among these eleven peptides the percentages of K/R range from 7.4% (D15) to 17% (D14). Plotting the ΔMW per amino acid residue against the percentages of K/R did not reveal a linear correlation (Fig. 5c). However, we cannot rule out the possibility of the effect of higher percentage of K/R on our equation.

### Successful prediction of the SDS PAGE-displayed MW for three acidic proteins Sas10, Mpp10 and Bms1l

**Figure 6:**
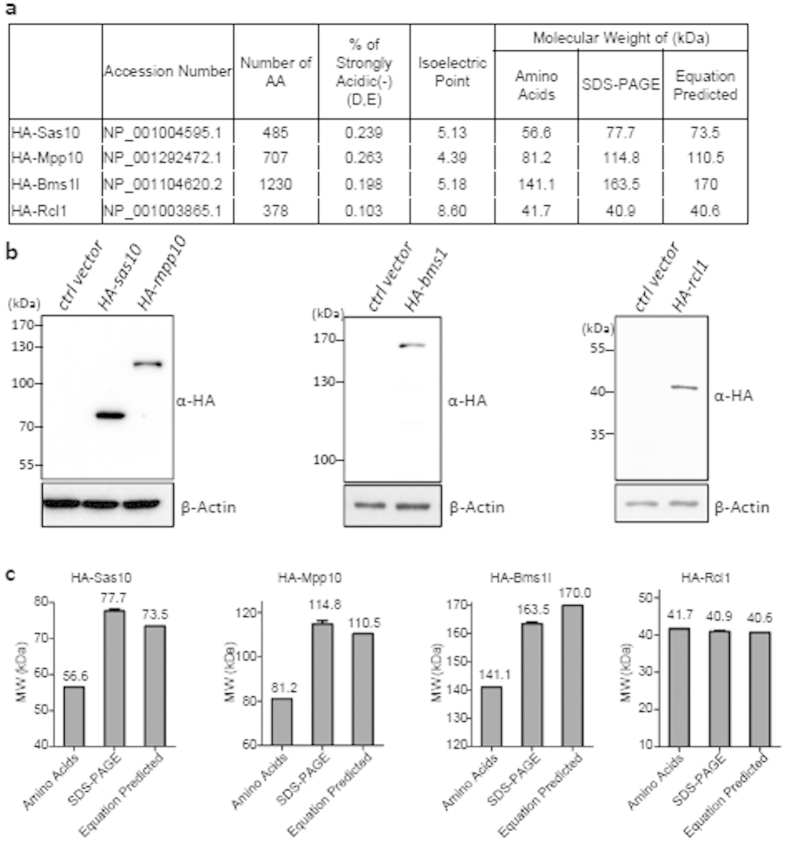
The equation is used to predict the SDS PAGE-displayed MWs for Sas10, Mpp10 and Bms1l successfully. (**a**) Summary of different indexes as listed for Sas10, Mpp10, Bms1l and Rcl1. (**b**) Western blot of HA-Sas10, and HA-Mpp10 (left panel), HA-Bms1l (middle panel) and HA-Rcl1 (right panel) with an HA antibody. (**c**) Showing the MW of calculated based on amino acids composition (Amino Acids), SDS PAGE-displayed (SDS-PAGE) and equation predicted for each protein. Number above indicates the MW. Error bars on the SDS PAGE-displayed column MW (mean) stands for SEM (n = 3). Western blot images were cropped with a grey cropping line. All gels for western blot analysis were run under the same experimental conditions.

Sas10^19^, Mpp10^13^ and Bms1l^20^,^21^ are all nucleolar acidic proteins. We cloned *sas10, mpp10* and *bms1l* into the expression vector pCS2^+^ with an HA tag and expressed them in the cultured human cells (293T), respectively. Western blot was used to determine the MW of these proteins in an SDS PAGE gel. HA-Sas10 is composed of 485 AA with a predicted isoelectric point of 5.13 (Fig. 6a). The theoretically predicted MW for HA-Sas10 is 56.6 kDa (Fig. 6a) and the actual SDS PAGE-displayed MW for HA-Sas10 is 77.7 kDa obtained by the western blot analysis (Fig. 6b, left panel). The actual SDS PAGE-displayed MW nicely matched the predicted SDS PAGE-displayed MW for HA-Sas10 (73.5 kDa) using the equation (Fig. 6c, panel for HA-Sas10). HA-Mpp10 is composed of 707 AA with a predicted MW of 81.2 kDa and isoelectric point of 4.39 (Fig. 6a). The actual SDS PAGE-displayed MW for HA-Mpp10 (114.8 kDa) (Fig. 6b, left panel) also nicely matched the predicted SDS PAGE-displayed MW (110.5 kDa) using the equation (Fig. 6c, panel for HA-Mpp10). HA-Bsm1l is composed of 1230 AA with a predicted MW of 141.1 kDa and isoelectric point of 5.18 (Fig. 6a). Similarly, the equation was successfully applied to predict the SDS PAGE-displayed MW for Bms1l (Fig. 6b, middle panel; 6c, panel for HA-Bms1l). As expected, the equation is also applicable to predict the SDS PAGE-displayed MW for Rcl1, a non-acidic protein (Fig. 6a–c, right panel in 6b, panel for HA-Rcl1 in 6c).

### Successful prediction of the SDS PAGE-displayed MW for ten acidic proteins reported in literatures

In order to further examine the applicability of the equation, we searched the literatures^10^,^22^,^23^,^24^,^25^,^26^,^27^,^28^,^29^,^30^ and got records for 10 acidic proteins with a percentage of E/D ranging from 18.8–31.2% (Table 1). As expected, the SDS PAGE-displayed MW for each of these ten proteins was larger than the predicted MW based on their amino acid composition as shown in the cited references (Table 1). We used the equation to estimate the SDS PAGE-displayed MWs for these ten proteins. The result clearly showed that the equation nicely predicted the SDS PAGE-displayed MW for each of these ten acidic proteins (Table 1).

## Discussion

It is not unusual to notice a protein displaying a MW on an SDS PAGE gel different from its predicted size (for example, while the predicted size for human p53 is 43.7 kDa it runs as a 53 kDa band in an SDS PAGE gel^31^,^32^). In many cases, this MW difference is attributed to chemical modifications of the protein, especially glycosylation and uibiquitination/sumoylation which causes drastically retarded gel mobility shift^16^,^17^. On the other hand, phosphorylation modification may causes subtle but significant band shift on an SDS PAGE gel^33^ except for hyper-phosphorylation which appears as a slower-migrating smear^34^. Therefore, there is a need to determine whether the larger size in an SDS PAGE gel is resulted from chemical modifications or from certain features (e.g AA composition) of the protein.

Def displayed an SDS PAGE MW approximately 13 kDa larger than its predicted one. To find out the reason behind, we carried out a series of peptide mapping experiments and found that the N-terminal 188 amino acid residues (2-189 AA) (D10 fragment) but not other regions of Def was responsible for its ~13 kDa mobility shift. We then used various approaches (including enzyme treatment and site-directed mutagenesis) to check whether the size difference was due to post-translational modification(s) of amino acid residue(s). We ruled out the contribution of glycosylation and unbiquitination/sumoylation to this MW difference. We did find that the first 95 amino acids at the N-terminus of Def was phosphorylated, however, that only accounts for a mere ~1.7 kDa mobility shift (data not shown), far less than the observed ~13 kDa MW difference for Def.

Eventually, we turned our focus on the AA composition of Def since the N-terminal region of Def (first 188 AA) contains a high percentage (35.6%) of acidic AA (E and D). We analyzed the relationship between MW of each of the thirteen peptides derived from Def with percentages of AA grouped based on their properties. We surprisingly found that the difference between the predicted and SDS PAGE-displayed MW for the Def N-terminus showed a linear correlation with the percentage of acidic AA (E and D) that fits the equation *y* = *276.5x* − *31.33* (where *x* represents the percentage of E and D, and *y* represents the average ΔMW per amino acid residue). Based on this formula we predicted that *y* (ΔMW) will be zero when *x* is 11.3%, in that case the observed MW on an SDS-PAGE gel would match the predicted MW. This was indeed the case for non-acidic protein Rcl1, a nucleolar protein with 10.3% of acidic AA (pI = 8.60). Finally, we demonstrated that this equation could be successfully applied to predict the SDS PAGE-displayed MW for thirteen acidic proteins, including Sas10, Mpp10 and Bms1l and ten others reported in the literatures. Therefore, this equation is practically useful because when a research encounters the MW difference issue it will allow us to predict the SDS PAGE-displayed MW conveniently prior to determining whether the MW difference is caused by chemical modifications of the protein of interest. Since the range of x value was deduced based on the lowest (Myc-D9) and highest (D16) percentages of E/D in the eleven peptides we tested (Fig. 5), it would be interesting to test whether our equation is applicable to predict the SDS PAGE-displayed MW for proteins with percentage of acidic amino acids beyond this range (lower or higher) in the future.

## Methods

### Zebrafish lines and maintenance

Zebrafish wild type AB line was maintained and used according to the standard procedures^35^. All animal procedures were performed in full accordance to the requirement by ‘Regulation for the Use of Experimental Animals in Zhejiang Province’. This work is approved by the Animal Ethics Committee in the School of Medicine, Zhejiang University (ETHICS CODE Permit NO. ZJU2011-1-11-009Y, issued by the Animal Ethics Committee in the School of Medicine, Zhejiang University).

### Morpholinos

*def*-MO and st-MO (the human β-globin antisense morpholino) were purchased from Gene Tools (LLC, USA) and used as described previously^1^.

### Plasmid construction

Target gene cDNA fragments (including *def* and its derivatives, *sas10, mpp10, rcl1* and *bms1l*) were cloned into the pCS2^+^ vector for *in vitro* mRNA synthesis. *myc* tagged *def, D1, D2, D3* and *D4* were constructed by Tao *et al.*^2^. The primers used for *myc* tagged *D9* and *D10* were listed in Supplementary Table 1.

To make the EGFP-D9 construct, primer pairs (*EGFP* Fw) + (*EGFP tag* Rv) and (*D9* Fw) + (*myc*-*D9* Rv) were used to amplify the EGFP and D9 respectively. PCR products were mixed and denatured together to allow annealing of the sticky ends to join the two parts, and this mixture was then used as the template for the second-round PCR using primers (*EGFP* Fw) and (*myc*-*D9* Rv) to get EGFP-D9. Similar method was used to obtain EGFP tagged D10, D15 and D17. EGFP tagged D14 and D16 were amplified from EGFP-D10 and EGFP-D15 respectively. The primers were listed in Supplementary Table 1.

All *def*-related mutant genes with point mutations were produced by site-directed mutagenesis PCR using the primers pairs listed in Supplementary Table 2.

### Plasmid transfection

293T cells were used for plasmid transfection. The cells were maintained in the DMEM medium (high glucose, GIBCO) supplemented with 10% fetal bovine serum (Gemini). Plasmids were transfected into the cell with Lipofectamine 2000 (Life Technologies) according to the manufacturer’s recommendations.

### mRNA synthesis and western blot

mRNAs were *in vitro* synthesized using mMESSAGE mMACHINE® Kit (Ambion) according to manufacturer’s instructions. mRNA was injected into one-cell stage zebrafish embryos to overexpress protein of interest. Embryos were deyolked and then lysed in SDS lysis buffer supplied with 1 × Complete Protease Inhibitor Cocktail (EDTA-free, Roche). The protein samples were used for western blot analysis immediately or kept at −20 °C for storage. Def rabbit polyclonal antibody used in western bolting was generated by Hangzhou HuaAn Biotechnology Company (China) using the synthesized peptide CLRLPDSPQRPEPDS. Anti-Myc tag antibody was purchased from Clontech (No. 631206). Sigma HA mouse monoclonal (HA-7) antibody (H3663) was used to detect HA tag. GFP Antibody (B-2) (Santa Cruz, sc-9996) was used to detect EGFP. β-Actin antibody was purchased from Cell Signaling (#4967). GAPDH rabbit monoclonal antibody (EPR1977Y) was from Epitomics (#5632-1).

### SDS PAGE-displayed MW calculation

For each SDS PAGE, prestained standard protein ladder (catalog number SM1811, a mixture of nine recombinant proteins with MWs of 11, 17, 28, 36, 55, 72, 95, 130 and 250 kDa, or SM0671, a mixture of ten recombinant proteins with MWs of 10, 15, 25, 35, 40, 55, 70, 100, 130 and 170 kDa, Fermentas) was loaded along with different protein samples. After gel electrophoresis, an R_f_ value (the migration distance of a protein divided by the migration distance of the front-running dye) was obtained for each standard control protein. The R_f_ value was plotted against the lg(MW) of corresponding standard control protein to get the linear formula lg(MW) = aR_f_ + b (where a is the slope and b is the y-intercept). The R_f_ value for each protein sample was then obtained and used for the calculation of the SDS PAGE-displayed MW of the corresponding protein.

### Glycosidase treatment

For glycosidase treatment deyolked embryos were lysed in 1X Glycoprotein Denaturing buffer and followed by heating at 95 °C for 5 minutes. 10 μl supernatant was made a total reaction volume of 20 μl by adding 2 μl 10X G7 Reaction buffer, 2 μl 10% NP40 and H_2_O. For N-deglycosylation 2 μl PNGase F (NEB, P0704) was added. For O-deglycosylation 2 μl O-glycosidase (NEB, P0733) and 2 μl Neuraminidase (NEB, P0720) were added. After incubation at 37 °C for 1 hour, reaction was stopped by adding 5 μl SDS sample buffer for analysis on an SDS-PAGE gel. RNase B (NEB, P7817S) and Fetuin (Sigma, F2379) were used as positive controls.

## Additional Information

**How to cite this article**: Guan, Y. *et al.* An equation to estimate the difference between theoretically predicted and SDS PAGE-displayed molecular weights for an acidic peptide. *Sci. Rep.* **5**, 13370; doi: 10.1038/srep13370 (2015).

## Supplementary Material

Supplementary Information

## Figures and Tables

**Table 1:** Successful prediction of the SDS PAGE-displayed MW for ten acidic proteins reported in literatures.

	Accessionnumber	Numberof AA	Percentageof D/E	Isoelectricpoint	Molecular Weight of (kDa)			Reference
					Aminoacidspredicted	SDS-PAGEdisplayed	Equationpredicted	
CKB1	NP_011496.3	278	0.209	4.41	32.2	38	39.5	22
Clc1p	NP_011683.3	233	0.275	4.15	26.5	38	36.9	23
EF1B	NP_009398.1	206	0.214	4.15	22.6	33	28.3	24
Enp1p	NP_009806.3	483	0.188	4.73	55.1	70	65.1	25
FPR3	NP_013637.1	411	0.287	4.24	46.6	65	66.4	26
HCP	P16230.1	852	0.312	4.55	96.1	165	143	10
Leo1p	NP_014766.1	464	0.287	4.19	53.9	80	76.1	27
Ltv1p	NP_012779.1	463	0.244	4.41	53.4	80	70.1	28
Mia40p	NP_012726.2	403	0.236	4.35	44.5	67.5	58.1	29
NAP1	NP_012974.1	417	0.237	4.1	47.9	60	62.2	30
