# Supplementary material for: An equation to estimate the difference between theoretically predicted and SDS PAGE-displayed molecular weights for an acidic peptide
Source: Sci Rep. 2015 Aug 27;5:13370. doi: 10.1038/srep13370 (PMC4550835; doi:10.1038/srep13370)
Supplement: Supplementary Information [file srep13370-s1.pdf]

An equation to estimate the difference between theoretically predicted and SDS PAGE-displayed molecular weights for an acidic peptide

Yihong Guan[1], Qinfang Zhu[1], Delai Huang[1], Shuyi Zhao[1], Li Jan Lo[1], and Jinrong Peng[1,*]

[1]MOE Key Laboratory for Molecular Animal Nutrition, College of Animal Sciences, Zhejiang University, 866 Yu Hang Tang Road, Hangzhou, China 310058

***:**Correspondence and requests for materials should be addressed to J.R.P (email: pengjr@zju.edu.cn).

Supplementary Table 1. PCR primers used for various cloning

Construct	Forward primer (5’-3’) (Fw)	Reverse primer (5’-3’) (Rv)
	myc* tagged *D9* and *D10	
myc-D9	CGCGGATCC**ATG**GAGGAGCAGAAGCTGATCTCAGAGGAGGACCTGCATGTGCAGCTGCCACATGTTTTTCAG	CGGAATTC**TCA**TGTGCTTTTCTCCTCCCCCGT
myc-D10	CGCGGATCC**ATG**GAGGAGCAGAAGCTGATCTCAGAGGAGGACCTGCATGGCAAAAGAAGGCGAGGAAAACAGGAG	CGGAATTC**TCA**CTGTTCCTGTTGTGCTGTGTTTTC
	EGFP* tagged *D9, D10, D14, D15, D16* and *D17	
EGFP	CGCGGATCC**ATG**GTGAGCAAGGGCGAGGAGCTG	CGCTCTAGA**TTA**CTTGTACAGCTCGTCCATGCC
EGFP tag	as “*EGFP*”	CTTGTACAGCTCGTCCATGCCGAG
D9	CTCGGCATGGACGAGCTGTACAAGGTGCAGCTGCCACATGTTTTT	as “*myc-D9*”
D10	CTCGGCATGGACGAGCTGTACAAGGGCAAAAGAAGGCGAGGAAAACAG	as “*myc-D10*”
D15	TCTCGGCATGGACGAGCTGTACAAGGATAACGAGGAGGAAGCTGAAGTTG	as “*D10*”
D17	TCTCGGCATGGACGAGCTGTACAAGGAAAAGACCAATGGAGATGTAGAG	as “*myc-D10*”
EGFP-D14	as “*EGFP*”	CGGAATTC**TCA**CTCTTCCTCGCTTTCTTCATCTTC
EGFP-D16	as “*EGFP*”	CGGAATTC**TCA**AGGGTGTTTTTTCTGTAATTTGTC
	HA tagged sas10, mpp10, bms1l and rcl1	
HA-sas10	GCGGATCCATGGAGTACCCATACGACGTACCAGATTACGCTCATATGGTCCGAGCAAGAAGGGCAGTAA	CGCTCGAGTCACTTGAGTTTGACACTTCTCTT
HA-mpp10	GCGGATCCATGGAGTACCCATACGACGTACCAGATTACGCTCATATGGCGACGAGGGATGGCGGCACG	GCGAATTCTTACAGCTTGAGTTTGTTCACAG
HA-bms1l	CGCGGATCCACCGCCATGTACCCATACGACGTACCAGATTACGCTGAGAGGAAAGAGCAGAAGCG	ACCGCTCGAGTCAATTGTCCTTGGATGTTCCCTT
HA-rcl1	CGCGGATCCATGGAGTACCCATACGACGTACCAGATTACGCTCATGAGTTTGAAGGTTGTAGCGGTTT	CGGAATTCTCATTTGATGCTTTTGCTGATGTTG

Supplementary Table 2. PCR primers used for site-direct mutagenesis		
**Construct**	**Forward primer (5'-3') (Fw)**	**Reverse primer (5'-3') (Rv)**
	**Mutated bases in lower case**	
K129R	GTTTTAGAGGATcgAGTGGAAGAAACA TCTGACAAATTACAGAAAAAACA	GTTTCTTCCACTcgATCCTCTAAAAC TTCCTCAGCATCATTCCCGTCCTC
K136R	AGAAACATCTGACcgATTACAGAAAAA ACACCCTGAAAAGACCAATGGAG	TTTTTCTGTAATcgGTCAGATGTTTCT TCCACTTTATCCTCTAAAACTTC
K139,140 R	GACAAATTACAGcgAcgACACCCTGAA AAGACCAATGGAGATGTAGAGGAGAA	TTTTCAGGGTGTcgTcgCTGTAATTTG TCAGATGTTTCTTCCACTTTATCCTC
K144R	AAACACCCTGAAcgGACCAATGGAGA TGTAGAGGAGAATGAAGAGGCAGA	TCTCCATTGGTCcgTTCAGGGTGTTT TTTCTGTAATTTGTCAGATGTTTC
K164,165 R	GAGTTTACAGATcgAcgGAATGAAGCTG CTTTCTGCTTGGAGACCAATATGCC	GCAGCTTCATTCcgTcgATCTGTAAAC TCTCCCTCCATCTCTGCCTCTTCATT
K136,139 ,140,144 R	cgATTACAGcgAcgACACCCTGAAcgGAC CAATGGAGATGTAGAGGAGAAT	cgTTCAGGGTGTcgTcgCTGTAATcgGT CAGATGTTTCTTCCACTcgATC
D14 K72R	GCATATCAGAgACTACTGTCCACCATG ATTCAAGGTGATGAAGATG	GGACAGTAGTcTCTGATATGCAGATG GTTGCTCAGCATCACTGTCA
D14 K34,41R	gGGTTGTTGAAAGACCGGAGAgGACT CAGATTTTGCGCTTGCCTGA	cTCTCCGGTCTTTCAACAACCcTATC GTGAAAAGGGTGTTGTTCAC
D14 K16,17,1 9,20,23R	TAgGAgACAGAgGAgACATCTAgAGGA ATTTGGTGAACAACACCCT	CTcTAGATGTcTCcTCTGTcTCcTAGTT AAATTATCAATCTCCTGT
D14 K3,8R	GCAgAAGAAGGCGAGGAAgACAGGA GATTGATAATTTAACTAgGAg	TGTcTTCCTCGCCTTCTTcTGCCCTTG TACAGCTCGTCCATGCCGA
